# Astrocyte Interactions With Ti_3_C_2_T_*x*_ MXene Flakes: Insights Into Viability, Morphology, and Functionality

**DOI:** 10.1002/admi.202500261

**Published:** 2025-08-18

**Authors:** Dimitris Boufidis, Elizabeth N. Krizman, Cybelle M. Smith, Yihan Xie, Raghav Garg, John C. O’Donnell, Flavia Vitale, D. Kacy Cullen

**Affiliations:** Department of Bioengineering, University of Pennsylvania, Philadelphia, Pennsylvania 19104, USA; Center for Neurotrauma, Neurodegeneration & Restoration, Corporal Michael J. Crescenz VA Medical Center, Philadelphia, Pennsylvania 19104, USA; Center for Neuroengineering and Therapeutics, University of Pennsylvania, Philadelphia, Pennsylvania 19104, USA; Center for Neurotrauma, Neurodegeneration & Restoration, Corporal Michael J. Crescenz VA Medical Center, Philadelphia, Pennsylvania 19104, USA; Department of Neurosurgery, University of Pennsylvania, Philadelphia, Pennsylvania 19104, USA; Center for Neurotrauma, Neurodegeneration & Restoration, Corporal Michael J. Crescenz VA Medical Center, Philadelphia, Pennsylvania 19104, USA; Department of Neurosurgery, University of Pennsylvania, Philadelphia, Pennsylvania 19104, USA; Department of Bioengineering, University of Pennsylvania, Philadelphia, Pennsylvania 19104, USA; Center for Neurotrauma, Neurodegeneration & Restoration, Corporal Michael J. Crescenz VA Medical Center, Philadelphia, Pennsylvania 19104, USA; Center for Neuroengineering and Therapeutics, University of Pennsylvania, Philadelphia, Pennsylvania 19104, USA; Department of Neurology, University of Pennsylvania, Philadelphia, Pennsylvania 19104, USA; Center for Neurotrauma, Neurodegeneration & Restoration, Corporal Michael J. Crescenz VA Medical Center, Philadelphia, Pennsylvania 19104, USA; Department of Bioengineering, University of Pennsylvania, Philadelphia, Pennsylvania 19104, USA; Center for Neurotrauma, Neurodegeneration & Restoration, Corporal Michael J. Crescenz VA Medical Center, Philadelphia, Pennsylvania 19104, USA; Center for Neuroengineering and Therapeutics, University of Pennsylvania, Philadelphia, Pennsylvania 19104, USA; Department of Neurology, University of Pennsylvania, Philadelphia, Pennsylvania 19104, USA; Department of Bioengineering, University of Pennsylvania, Philadelphia, Pennsylvania 19104, USA; Center for Neurotrauma, Neurodegeneration & Restoration, Corporal Michael J. Crescenz VA Medical Center, Philadelphia, Pennsylvania 19104, USA; Center for Neuroengineering and Therapeutics, University of Pennsylvania, Philadelphia, Pennsylvania 19104, USA; Department of Neurosurgery, University of Pennsylvania, Philadelphia, Pennsylvania 19104, USA

**Keywords:** astrocytes, calcium imaging, morphology, neural interfaces, Ti_3_C_2_T_x_ MXene, viability

## Abstract

Astrocytes, the most abundant cell type in the brain, are increasingly recognized as active regulators of neuronal function and potential therapeutic targets. Two-dimensional MXenes, such as titanium carbide (Ti_3_C_2_T_x_), hold promise for neural interfaces and bioelectronic applications owing to their favorable electrochemical properties, but their compatibility with glial cells has yet to be investigated. Here, the first systematic evaluation of astrocyte–MXene interactions is reported by exposing cortical rat astrocytes to varying concentrations of Ti_3_C_2_T_x_ in aqueous dispersions. Phase contrast and scanning electron microscopy showed individual flakes and larger aggregates on astrocyte membranes with no detectable damage. Cytotoxicity assays demonstrated robust astrocyte viability, morphological analysis indicated no significant changes in cell structure, and calcium imaging revealed no changes in spontaneous Ca^2+^ activity between control and Ti_3_C_2_T_x_-treated cultures. These findings establish a foundation for the integration of Ti_3_C_2_T_x_ into next-generation neural interfaces while underscoring the need for further exploration of MXene-based tools for astrocyte-targeted neuromodulation.

## 1. Introduction

The brain is a complex organ comprising billions of specialized cells, including neurons and glial cells. While neurons are the primary signaling units of the nervous system, glial cells -a diverse group of cells that include astrocytes, oligodendrocytes, and microglia- are essential for supporting neuronal function, maintaining structural integrity, and responding to injury.^[1–3]^ Astrocytes, the most abundant cell type in the brain, actively regulate neurotransmitter uptake, modulate synaptic activity, maintain the blood-brain barrier, and provide neurotrophic support.^[1,2,4,5]^ During injury, astrocytes play a critical role in repair mechanisms by forming glial scars and modulating inflammatory signaling.^[1,6,7]^ Historically dismissed as passive support cells, merely the “glue” of the nervous system, astrocytes are now recognized as active regulators of synapse formation, plasticity, and overall brain health and function.^[1,2,8]^ Calcium signaling is a key component of astrocyte function, with intracellular calcium ion fluctuations regulating diverse astrocytic functions and cellular processes.^[1]^ Aberrations in astrocyte calcium signaling have been implicated in numerous neurological disorders, including Alzheimer’s disease, epilepsy, Huntington’s and Parkinson’s diseases, autism spectrum disorders, and various cognitive and neuropsychiatric conditions.^[6,9–13]^ As such, innovative strategies to control and modulate astrocyte function could create powerful therapeutic opportunities for addressing otherwise intractable neuropathologies and dysfunction.

Neuroelectronic technologies are transforming the clinical diagnosis and treatment of neurological conditions, from restoring motor function after paralysis to decoding speech and alleviating Parkinson’s disease symptoms.^[14–17]^ However, seamlessly integrating these interfaces with neural tissue remains a significant challenge.^[18]^ The foreign body response (FBR) to implanted materials can trigger inflammation, astrogliosis, and device encapsulation, ultimately impairing long-term functionality.^[19,20]^ Astrocytes, as key regulators of neuroinflammation and scar formation, play a central role in the FBR, making their response to neuroelectronic materials a critical determinant of device performance.^[20]^ Additionally, astrocytes are increasingly recognized as direct targets for neuromodulation, with recent studies exploring astrocyte stimulation to influence neuronal activity.^[20,21]^ As such, novel bioelectronic materials must not only ensure neuronal compatibility but also establish how they interact with astrocytes to enhance the stability and effectiveness of next-generation neural interfaces.

Two-dimensional (2D) nanomaterials, such as MXenes, have gained significant attention for their potential in next-generation neural interfaces and targeted stimulation.^[21,22]^ MXenes are a class of transition metal carbides and nitrides with high electronic conductivity, large surface area, and tunable surface chemistry, making them highly attractive for biomedical applications.^[23]^ Among them, titanium carbide Ti_3_C_2_T_x_ has been successfully demonstrated in neural microelectrodes,^[24]^ remote non-genetic optical neurostimulation,^[22,25]^ as well as modulation of neuronal maturation, growth, and differentiation.^[26]^ Given their potential for neuronal applications, we hypothesize that astrocyte–MXene interfaces may also enable selective modulation of astrocytic activity, advancing the emerging field of glial engineering.^[21,27]^ Biocompatibility is a fundamental requirement for MXenes and any other new material used to interface with cells and tissues. Previous studies have shown the viability and growth of neurons exposed to Ti_3_C_2_T_x_ in vitro, with some dose-dependent cytotoxic effects at concentrations >25 μg mL^−1^.^[24,28,29]^ Similarly, prior studies have demonstrated the immunocompatibility of Ti_3_C_2_T_x_ MXene flakes with macrophages and other immune cells.^[30]^ Yet, despite astrocytes’ abundance and critical role in the central nervous system, the impact of Ti_3_C_2_T_x_ MXene on astrocytes remains unknown.^[31]^ Given the unique geometry, high surface-area-to-thickness ratio, and broad lateral size distribution of Ti_3_C_2_T_x_ flakes, we hypothesized that these 2D nanomaterials could engage in physical interactions with astrocytes, which may influence astrocyte structure or function.

**Scheme 1.**
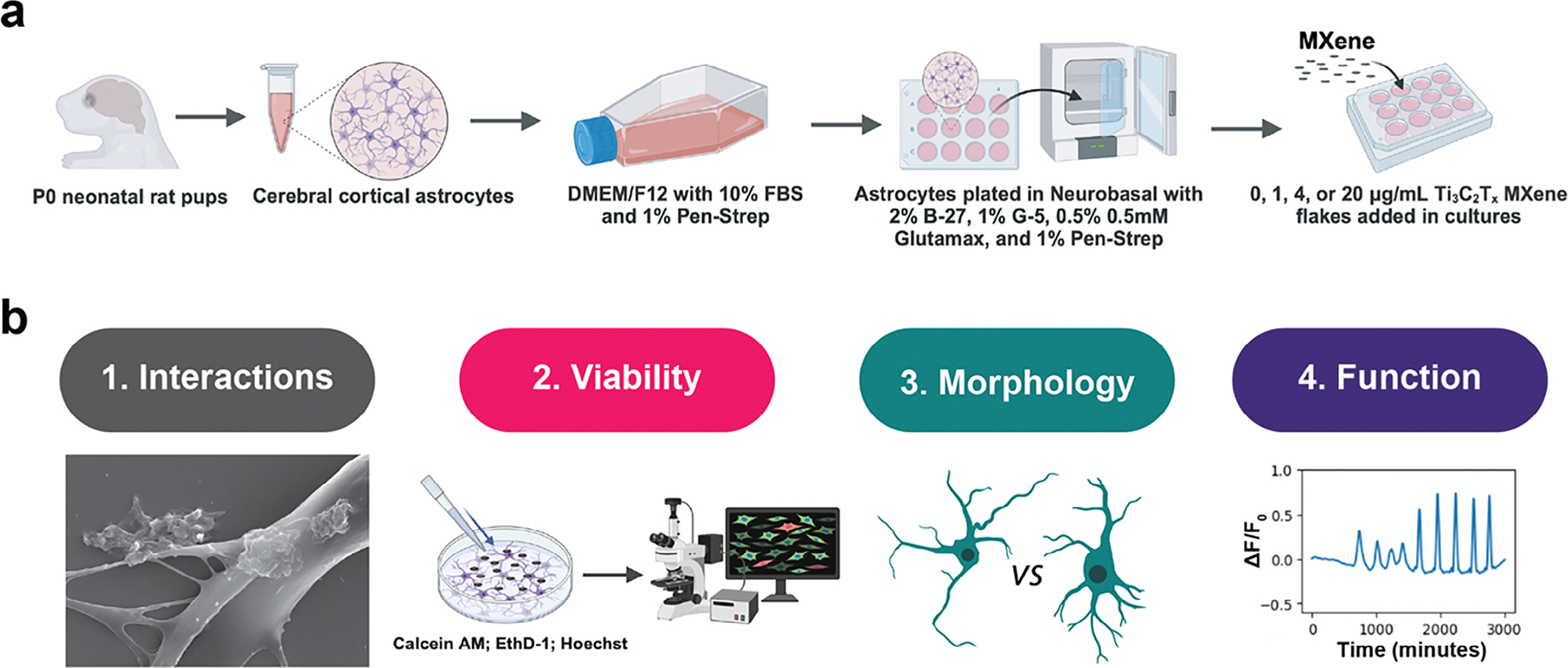
a) Schematic illustration with an overview and key experimental methods of the study. b) Overview of the four major metrics of analysis: (1) astrocyte–MXene interactions, (2) cell viability, (3) morphology, and (4) functionality.

This study provides the first systematic evaluation of interactions between astrocytes and Ti_3_C_2_T_x_ MXene, as illustrated in Scheme 1 Specifically, rat astrocytes were treated with increasing concentrations of Ti_3_C_2_T_x_ MXene flakes, and we investigate four major metrics of analysis: (1) astrocyte–MXene adhesion and interactions, (2) cell viability, (3) morphological changes, and (4) intrinsic calcium signaling.

## 2. Results and Discussion

### 2.1. Interaction of Ti_3_C_2_T_x_ MXenes with Astrocytes

**Figure 1.**
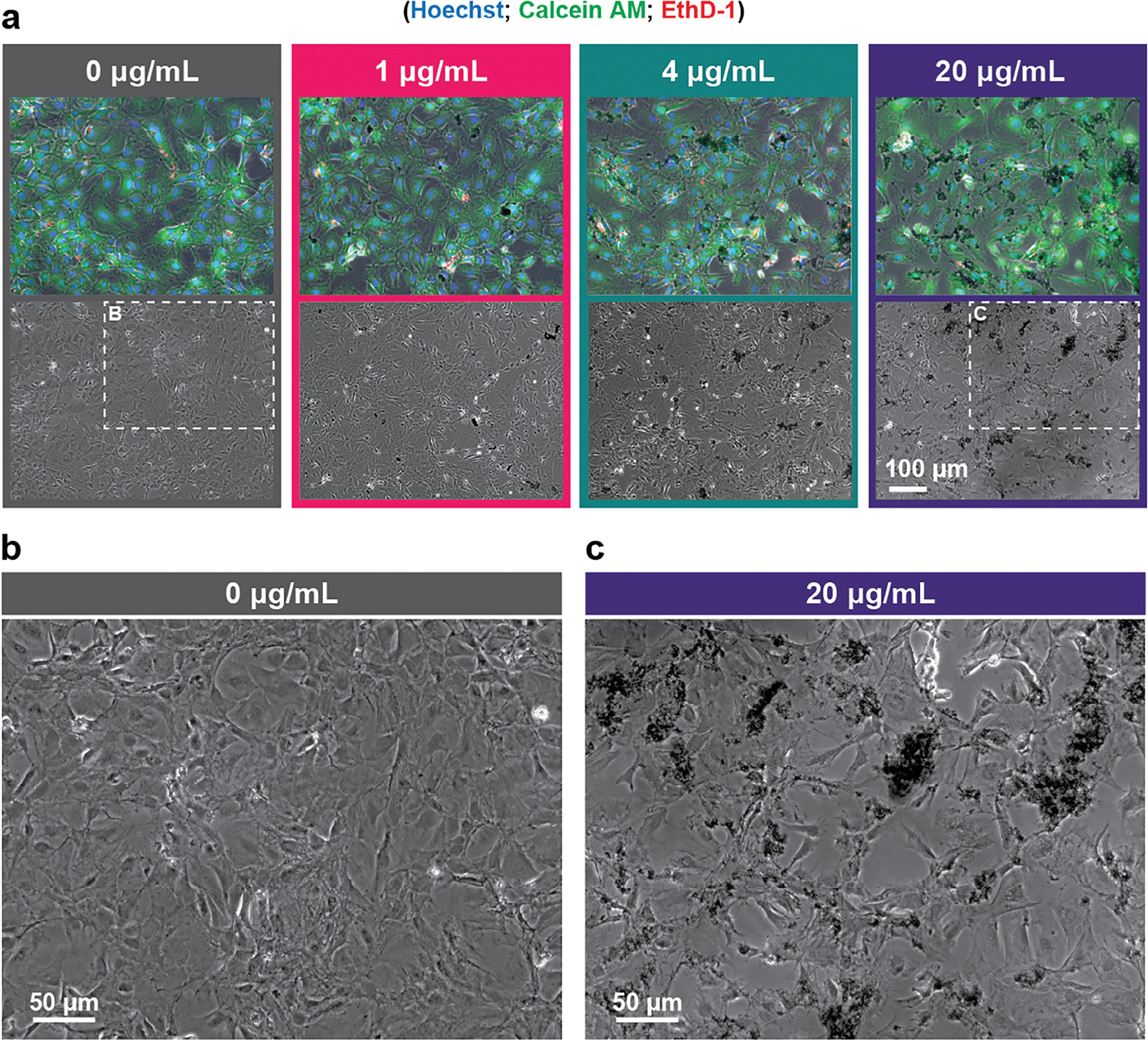
Astrocytes cultured for 10 days in vitro (DIV) with Ti_3_C_2_T_x_ MXene at different concentrations. a) Representative fluorescence (top) and phase contrast (bottom) images of astrocytes exposed to Ti_3_C_2_T_x_ flakes at 0, 1, 4, and 20 μg mL^−1^ concentrations. Ti_3_C_2_T_x_ flakes and aggregates appear black and become more prominent at higher concentrations. Fluorescence channels show Calcein-AM (green), Ethidium Homodimer-1 (red), and Hoechst (blue). b,c) Zoomed-in phase contrast images of (b) untreated cells and (c) 20 μg mL^−1^ Ti_3_C_2_T_x_-treated astrocytes from the region marked with the white rectangles in (a).

This study provides the first detailed visualization of Ti_3_C_2_T_x_ MXene flakes in astrocyte cultures in vitro. Primary cerebral cortical astrocytes from neonatal rat pups were cultured in vitro before adding Ti_3_C_2_T_x_ MXene flakes at 0, 1, 4, and 20 μg mL^−1^ concentrations. Dynamic light scattering (DLS) analysis revealed a mean particle lateral size of 1.3 μm, and Raman spectroscopy confirmed the structural and chemical composition of the MXene flakes, with characteristic peaks consistent with previously reported signatures of Ti_3_C_2_T_x_^[32,33]^ (Figure S1, Supporting Information). Phase contrast images reveal Ti_3_C_2_T_x_ flakes distributed across the astrocyte culture (Figure 1a). These flakes become increasingly evident with increasing concentrations of Ti_3_C_2_T_x_ MXene (1, 4, and 20 μgmL^−1^), while they are absent in untreated cultures. Both individual flakes and larger aggregates are observed, consistent with previous reports of Ti_3_C_2_T_x_ in ionic solutions.^[34,35]^ The formation of these multi-flake aggregates is primarily attributed to the negatively charged surface terminations (T_x_) of Ti_3_C_2_T_x_, which facilitate electrostatic interactions with ions in the culture medium, compressing the electrical double layer and promoting flake clustering.^[34,35]^ Neurobasal medium, containing 51.7 mm NaCl, among other salts, creates a high ionic strength environment conducive to aggregation.^[34,36]^ Zoomed-in phase contrast images further highlight the differences between untreated astrocytes (Figure 1b) and those exposed to 20 μg mL^−1^ Ti_3_C_2_T_x_ (Figure 1c). In the treated cultures, dense Ti_3_C_2_T_x_ MXene flakes and aggregates cover portions of the astrocyte monolayer, contrasting with the smooth and uniform appearance of untreated astrocyte cultures. However, despite the visible accumulation of MXenes, fluorescence imaging indicates that these aggregates do not consistently colocalize with dead cells (Ethidium Homodimer-1, red), suggesting minimal localized cytotoxicity. While the 20 μg mL^−1^ dose ensured an even distribution of flakes and aggregates across the entire astrocyte monolayer, higher MXene concentrations result in a dense, saturated layer that may obscure cellular features (Figure S2, Supporting Information).

### 2.2. High-Resolution Imaging of Ti_3_C_2_T_x_ MXene Flakes on Astrocytes

**Figure 2.**
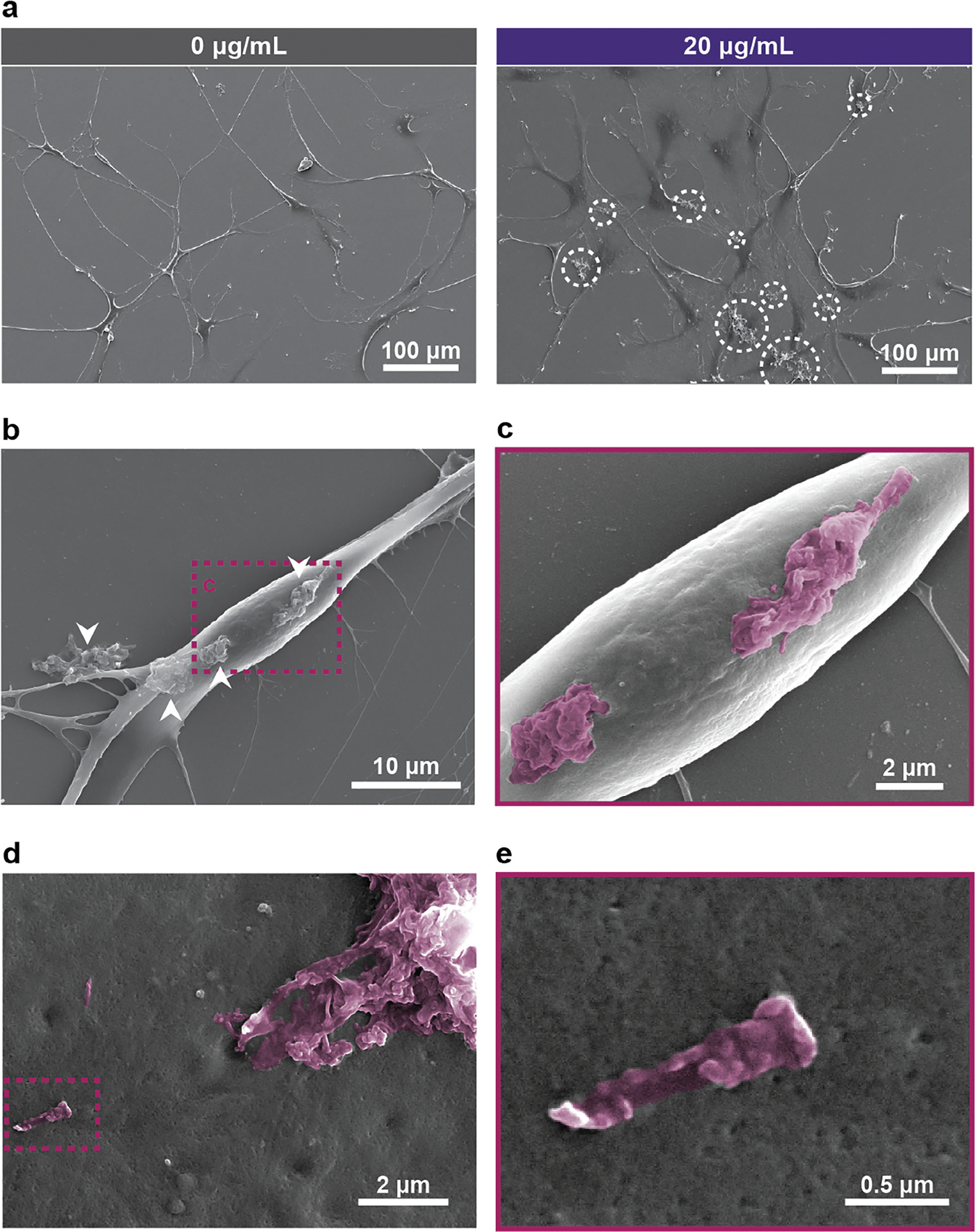
Scanning Electron Microscopy (SEM) of astrocytes cultured with Ti_3_C_2_T_x_ for 10 days in vitro. a) Representative 200 × SEM images showing untreated (0 μg mL^−1^, left) and Ti_3_C_2_T_x_-exposed (20 μg mL^−1^, right) astrocytes. White circles highlight Ti_3_C_2_T_x_ flakes and aggregates. b) SEM at 2500 × magnification showing Ti3C2Tx flakes firmly adhering to the astrocyte membranes. c) Zoomed-in SEM at 5000 × of the highlighted region in (b). SEM micrograph of d) 5000 × and e) 20000 × magnification of Ti_3_C_2_T_x_ on the astrocyte membrane. Ti_3_C_2_T_x_ is digitally colored at (c–e) to enhance contrast and visibility (magenta).

Scanning electron microscopy (SEM) imaging provides additional insights into the interaction between Ti_3_C_2_T_x_ MXene flakes and astrocytes in vitro. At 200x magnification, SEM shows distinct aggregates distributed across the cell surface of Ti_3_C_2_T_x_-treated cultures, which were absent in untreated astrocytes (Figure 2a). At higher magnifications (2500 ×), Ti_3_C_2_T_x_ aggregates are firmly adhering to the astrocyte membrane (Figure 2b), with zoomed-in SEM images (5000 ×) revealing fine structural details (Figure 2c). Both individual flakes (≈1 μm lateral dimension) and larger aggregates are visible, indicating the heterogeneous size distribution of Ti_3_C_2_T_x_ within the culture (Figure 2d,e). At 20000 × magnification, the nanoscale morphology of flakes becomes evident, revealing sharp edges and rough surface topology (Figure 2e). Despite the close adherence of Ti_3_C_2_T_x_ flakes to the astrocyte membrane (Figure 2b–e, Figure S3, Supporting Information), there are no apparent signs of membrane damage or localized toxicity. In some cases, astrocytic processes were observed extending toward and contacting large Ti_3_C_2_T_x_ aggregates that were not attached to the cell membrane (Figure S4, Supporting Information). The adhesion of Ti_3_C_2_T_x_ MXene flakes to cell membranes demonstrates their potential for stable cell-material interfaces across various biomedical applications.

### 2.3. Astrocyte Viability Following Ti_3_C_2_T_x_ MXene Exposure

**Figure 3.**
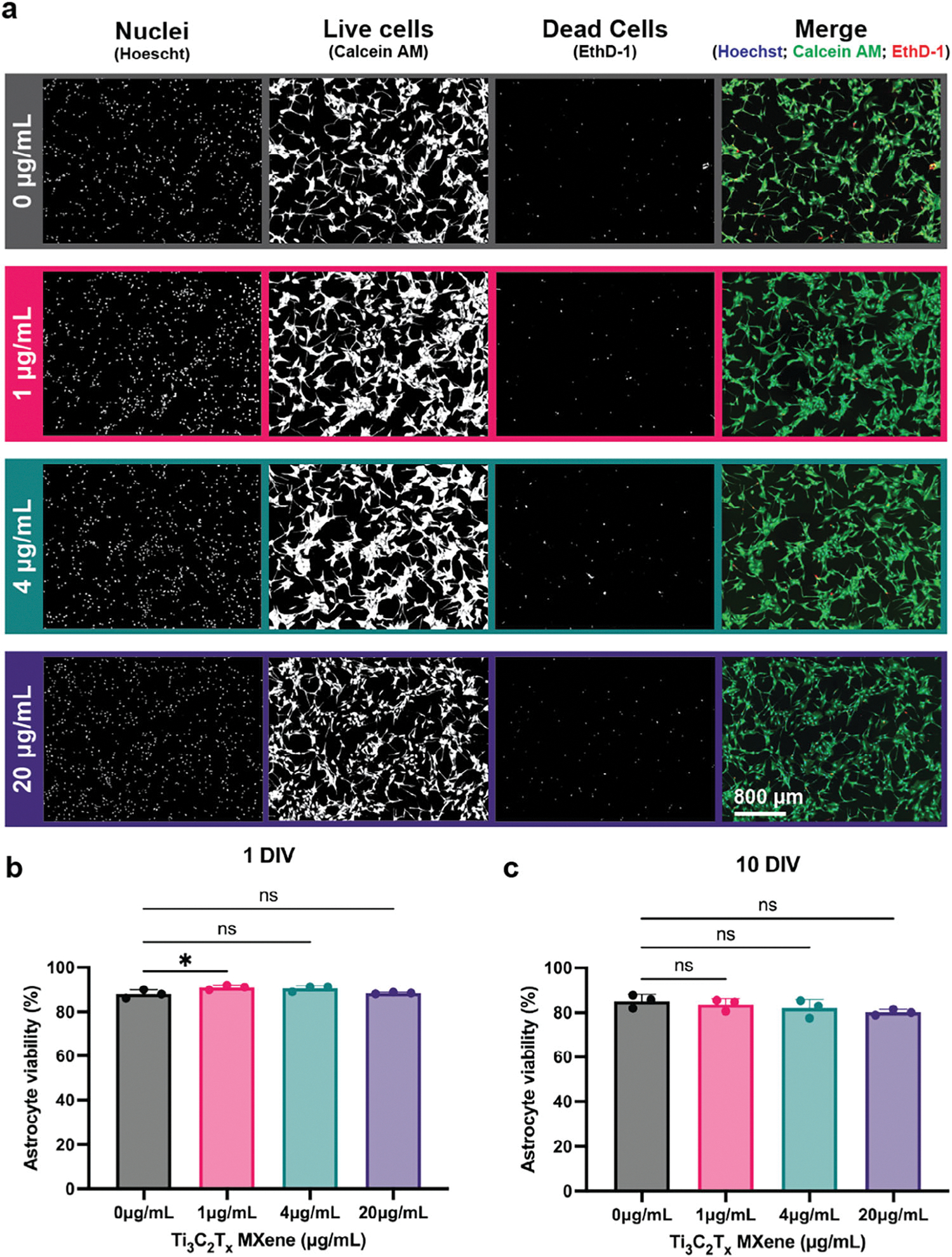
Astrocyte viability following exposure to varying concentrations of Ti_3_C_2_T_x_ MXene at 1 and 10 days. a) Representative fluorescent microscopy images of astrocytes treated with 0, 1, 4, and 20 μg mL^−1^ Ti_3_C_2_T_x_. Individual channels are shown as white on a black background for maximum contrast, and overlays of the fluorescent images merge all channels (Calcein AM: Green, EthD-1: red, Hoechst: blue). b,c) Quantification of astrocyte viability at (b) 1 and (c) 10 days in vitro (DIV) shows high viability across all time points and treatment groups. Data are represented as mean ± SEM. *n* = 3 biological replicates per condition, each with 3 technical replicates and ≥4 fields per replicate. Asterisks (*) denote statistically significant difference with p < 0.05 (one-way ANOVA and post-hoc Tukey).

Having established that Ti_3_C_2_T_x_ MXene flakes adhere to astrocyte membranes without apparent membrane disruption or localized toxicity, we next quantified astrocyte viability to determine whether Ti_3_C_2_T_x_ exposure induces dose- and/or time-dependent cytotoxic effects. For this analysis, we used a live/dead fluorescence assay, combining three markers: Calcein-AM (green), which stains live cells based on intracellular esterase activity; Ethidium Homodimer-1 (red), which labels dead cells by binding to nucleic acids in cells with compromised membranes; and Hoechst (blue), which stains cell nuclei and identifies the total cell population. Fluorescence microscopy images show robust astrocyte viability at all tested concentrations of Ti_3_C_2_T_x_ (0, 1, 4, and 20 μg mL^−1^) for both 1 and 10 days in vitro (DIV), with Hoechst staining indicating comparable cell density across all treatment groups. (Figure 3a). Quantitative analysis corroborated these observations, revealing consistently high astrocyte viability despite increasing concentrations of MXene (Figure 3b,c). At 1 DIV, astrocyte viability remained high across all conditions, with mean percentage viability ± standard deviation of 88.1 ± 1.9% (0 μg mL^−1^), 91.0 ± 1.0% (1 μg mL^−1^), 90.5 ± 1.3% (4 μg mL^−1^), and 88.5 ± 0.4% (20 μg mL^−1^). A slight increase in viability was observed at the lowest dose (1 μg mL^−1^) compared to untreated controls (*p* < 0.05). By 10 DIV, viability remained above 80% in all groups, with values of 85.2 ± 3.0% (0 μg mL^−1^), 83.6 ± 2.6% (1 μg mL^−1^), 82.0 ± 4.0% (4 μg mL^−1^), and 80.2 ± 1.3% (20 μg mL^−1^). No statistically significant differences were observed between control and Ti_3_C_2_T_x_-treated cells at this time point, indicating minimal cytotoxic effects even with prolonged MXene exposure (10 DIV). Although our results align with earlier studies indicating minimal MXene toxicity to neurons and neural stem cells^[24,28,29]^ a subtle dose-dependent reduction in viability was noted at 10 DIV. This trend may suggest subtle or delayed effects of prolonged Ti_3_C_2_T_x_ exposure, warranting future investigation with extended timepoints. However, the probability that this trend is due to random chance is rather high and did not reach statistical significance (*p* = 0.1504).

While this study offers the first insights into astrocyte–Ti_3_C_2_T_x_ interactions, additional research is needed to elucidate the fate of MXene flakes, including potential uptake by astrocytes and the long-term implications of such interactions. Future studies could include additional assays, such as metabolic activity assays (e.g., MTT), membrane integrity tests (e.g., LDH release), reactive oxygen species (ROS) quantification, and gene expression profiling.^[28,37]^ Surface modifications, such as biopolymer coatings, have also been shown to reduce residual cytotoxicity and improve MXene stability in physiological environments.^[37]^ For instance, laminin-coated Ti_3_C_2_T_x_ films promote neural stem cell adhesion, proliferation, and differentiation^[38]^ and have been used to support the growth and maturation of primary neuron cultures.^[26]^ However, direct comparison between MXene flakes and films cannot be directly drawn, given the effects of film topologies and surface chemistries on the cell-film interactions. Films are typically used as growth substrates that support basal adhesion and cell contact, whereas MXene flakes introduced after cell attachment primarily interact with the apical surface of cells. Future studies will be needed to elucidate these chemistry and topology-dependent effects on cellular processes.

### 2.4. Astrocyte Morphology Analysis

**Figure 4.**
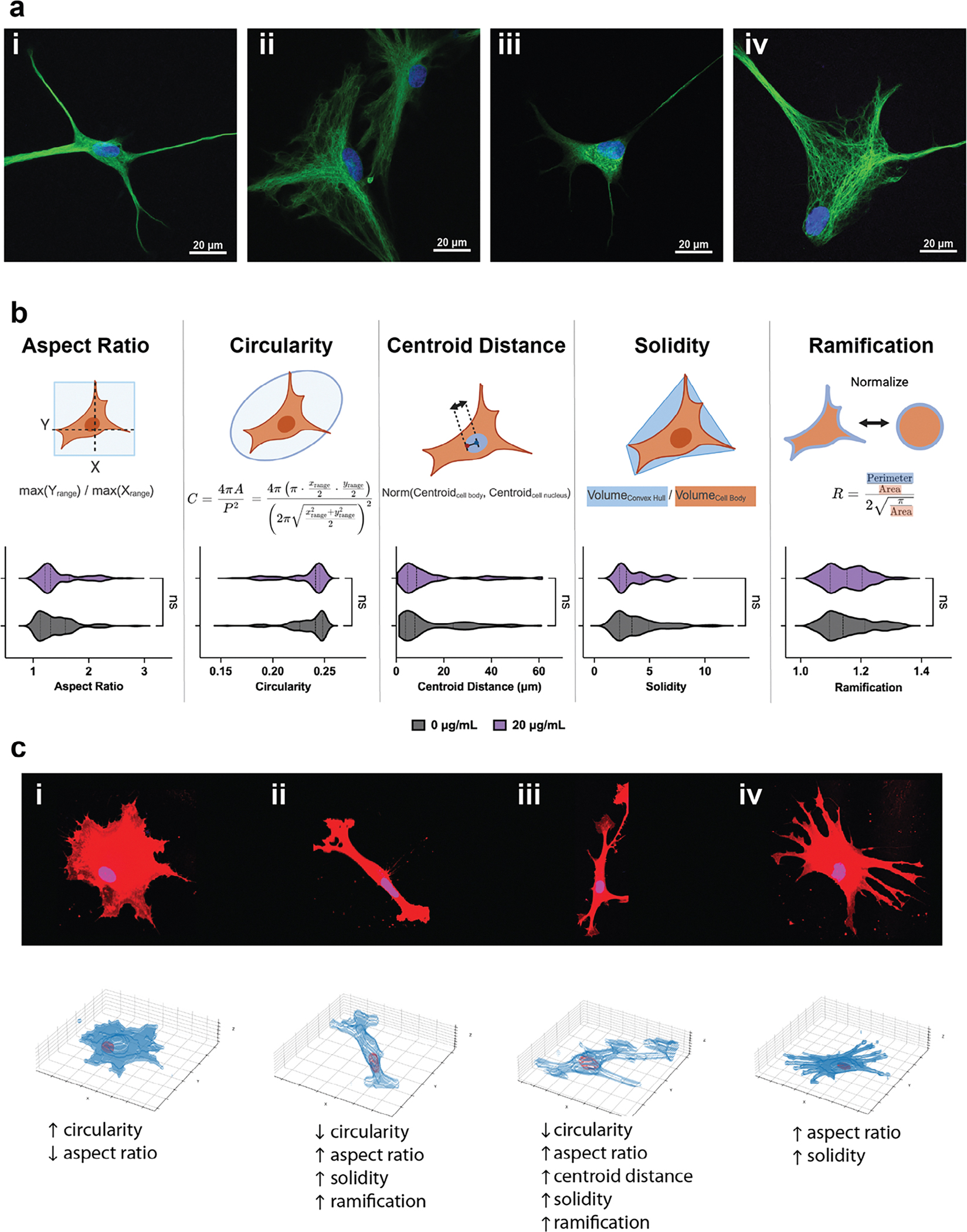
a) Confocal microscopy images (100 × magnification) illustrating the heterogeneous astrocyte morphology. (i, ii) Control and (iii, iv) Ti_3_C_2_T_x_-treated astrocytes for 2 days in vitro exhibit GFAP-positive filamentous structures with varied morphologies across both conditions. b) Schematics illustrate the key morphological parameters analyzed (aspect ratio, circularity, centroid distance, solidity, ramification), alongside violin plots depicting their distributions for astrocytes treated with 0 and 20 μg mL^−1^ Ti_3_C_2_T_x_ for 1 day in vitro. Statistical comparisons of morphological parameters were performed using a two-tailed unpaired t-test, with statistical significance defined as p < 0.05. n = 45 images per group. C) Representative examples of confocal images and corresponding 3D reconstructions highlight astrocytes with low and high values of morphological parameters. Fluorescence images are maximum-intensity projections of cells immunostained with plasma membrane markers, while 3D reconstructions represent the output of the morphology analysis pipeline. Up arrows (↑) represent high values (top 33% for any given morphological parameter), while down arrows (↓) represent low values (bottom 33%). Only parameters falling within the highest or lowest tertile were flagged as high or low.

Building upon our viability findings, we next evaluated astrocyte morphology to determine whether Ti_3_C_2_T_x_ MXene flakes influenced cellular shape and function. The inherent heterogeneity of astrocyte morphology should be carefully considered when interpreting morphometric data, as overlapping distributions in metrics can obscure subtle functional differences. Indeed, high-magnification (100x) confocal imaging of GFAP-stained astrocytes demonstrated a broad spectrum of morphological diversity, ranging from circular to highly branched, irrespective of the treatment groups (Figure 4a). This morphological diversity of GFAP-positive astrocytes was observed across all experimental conditions, indicating that the presence of Ti_3_C_2_T_x_ flakes did not alter the baseline heterogeneity characteristic of astrocytes cultured in vitro. These findings corroborate previous studies describing planar astrocytes with diverse arrangements of intermediate filaments.^[39]^ Here, we analyzed astrocytes cultured with 0 and 20 μg /mL^−1^ Ti_3_C_2_T_x_ for 1 day in vitro using a custom image analysis pipeline (Figure S5, Supporting Information). Specifically, we quantified several morphological parameters such as aspect ratio, circularity, centroid distance, solidity, and ramification, as detailed in the Methods section. Violin plots illustrate the distribution of these metrics across conditions, with no significant differences (*p* = ns) in astrocyte morphology upon Ti_3_C_2_T_x_ MXene exposure (Figure 4b). The aspect ratio, which reflects cell elongation, remained comparable between conditions (0 μg mL^−1^: 1.40 ± 0.34, 20 μg mL^−1^: 1.48 ± 0.41). Similarly, circularity, a measure of how close a shape is to a perfect circle, did not significantly differ (0 μg mL^−1^: 0.23 ± 0.02, 20 μg mL^−1^: 0.23 ± 0.02). Centroid distance, which measures the displacement between the nucleus and the geometric center of the cell body, remained consistent across conditions (0 μg mL^−1^: 14.00 ± 13.96, 20 μg mL^−1^: 14.75 ± 15.55) but exhibited substantial variability in both groups. This variability likely reflects the natural heterogeneity of astrocyte morphology, as well as intrinsic differences in cell migration and division. Solidity, a parameter representing cell density relative to its convex hull, also showed no significant changes (0 μg mL^−1^: 3.90 ± 2.15, 20 μg mL^−1^: 3.46 ± 1.51). Finally, ramification, a metric assessing the degree of complexity of the cell shape, remained unchanged (0 μg mL^−1^: 1.16 ± 0.08, 20 μg mL^−1^: 1.15 ± 0.07). These findings suggest that Ti_3_C_2_T_x_ exposure does not alter astrocyte morphology under the tested conditions, supporting the structural resilience of astrocytes in vitro. This aligns with morphometric studies of microglia, where cellular shape metrics correlate with a spectrum of functional states but often require substantial stimuli to elicit morphological shifts.^[40,41]^ Representative examples of confocal images and their corresponding 3D reconstructions of cells contextualize the metrics with visual interpretations (Figure 4c). For instance, low circularity corresponds to highly elongated astrocytes, while low solidity and ramification indicate less complex cell body geometries. Similarly, a higher centroid distance indicates cells where the nucleus centroid is further displaced from the cell body’s center of mass. While no single metric can definitively classify astrocyte reactivity, changes in morphological descriptors such as aspect ratio, circularity, solidity, and ramification have been reported as astrocyte responses to inflammation, nanomaterial exposure, and matrix stiffness. For example, lower circularity and solidity have been associated with more stellate, process-bearing morphologies in models of injury and reactive gliosis^[42,43]^ whereas increased circularity and solidity may reflect cellular hypertrophy or swelling under metabolic stress conditions.^[42]^ Increased ramification has been observed in astrocytes cultured on soft, injury-mimicking hydrogels compared to baseline-stiffness substrates.^[44]^ Changes in aspect ratio have also been linked to elongated morphologies often observed in reactive astrocytes.^[45–47]^ Nonetheless, given the variability of in vitro astrocyte morphology and the absence of a universal morphometric signature for reactive states, we interpret these results as indicating that Ti_3_C_2_T_x_ exposure did not grossly alter the morphology of astrocytes under the culture conditions used in this study. We also acknowledge the limitations of 2D cultures in vitro, in which astrocytes lack the 3D mechanical and biochemical cues present in vivo that may influence their morphology and interactions with nanomaterials like Ti_3_C_2_T_x_ MXene.

### 2.5. Calcium Imaging

**Figure 5.**
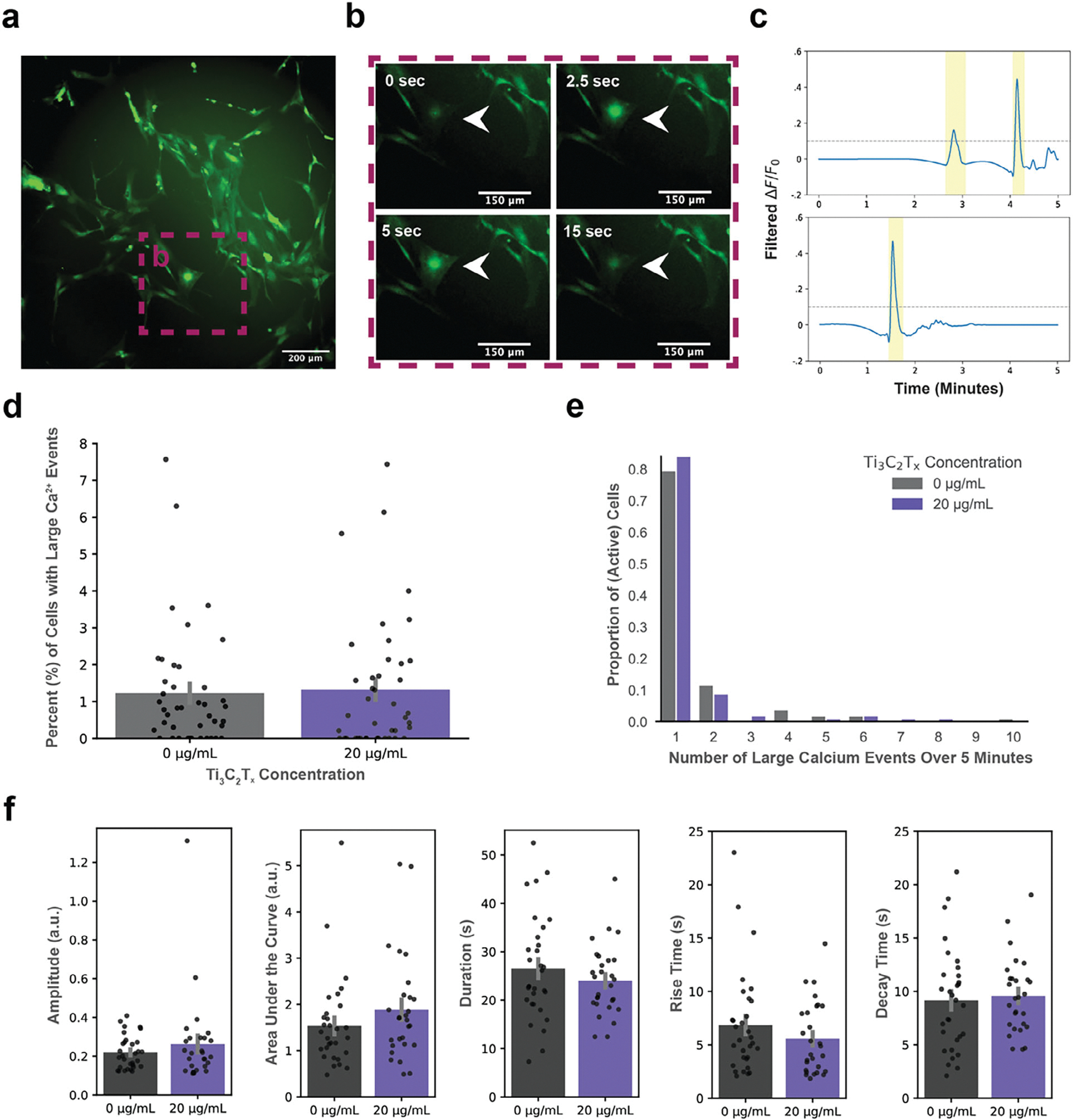
Spontaneous calcium activity of astrocytes upon Ti_3_C_2_T_x_ MXene exposure. a) Maximum intensity projection of a 5-min time-lapse calcium imaging video from Calbryte 520-labeled astrocytes. b) Selected frames from a zoomed region of interest (ROI) highlighted in (a), illustrating a representative large-scale spontaneous calcium event. c) Representative ΔF/F_*n*_ calcium traces from individual astrocytes in control (top) and Ti_3_C_2_T_x_-treated (20 μg mL^−1^, bottom) conditions. Yellow shading denotes detected large events. d) Quantification of the percentage of cells exhibiting at least one large calcium event over the 5-min imaging period, n = 42 videos per condition. e) Distribution of the number of large calcium events per active cell in each condition. f) Quantification of calcium event kinetics in active cells, including amplitude, area under the curve (AUC), event duration, rise time, and decay time. No statistically significant differences (p < 0.05) were observed between control and MXene-treated groups across any metric. Data are shown as mean ± SEM.

To evaluate whether exposure to Ti_3_C_2_T_x_ MXene flakes alters astrocyte function, we acquired time-lapse calcium imaging using Calbryte 520 AM to monitor spontaneous intracellular Ca^2+^ dynamics in control and Ti_3_C_2_T_x_-treated (20 μg mL^−1^) astrocyte cultures. A representative maximum projection image is shown in Figure 5a, with a zoomed region highlighting a spontaneous large-scale calcium transient (Figure 5b). A binary segmentation mask was created from the maximum projection fluorescence image, and calcium traces were extracted from regions of interest (ROIs) corresponding to individual cells (Figure S6, Supporting Information). Large-scale calcium events were identified using a threshold-based detection algorithm (Figure 5c). Imaging was performed on 42 independent wells per condition, each recorded for 5 min, resulting in a total of 84 unique calcium imaging videos. Experimental and acquisition parameters are described in the Methods. Quantification showed no significant difference in the proportion of cells exhibiting large calcium events between conditions (Student’s two-sample t-test by well: t(82) = .265; Figure 5d), and the number of events per active cell followed similar distributions in both groups (Kolmogorov-Smirnov test: KS = .045; Figure 5e). To further assess potential functional changes, we quantified calcium event kinetics (amplitude, area under the curve, duration, rise time, and decay time) in all active cells. These parameters are important because they can regulate gliotransmitter release and thereby influence cellular function.^[48]^ We did not observe significant differences between MXene-exposed and control astrocytes (Student’s two-sample t-test; amplitude: 0 μg mL^−1^ mean = 0.262, 20 μg mL^−1^ mean = 0.220, t(57) = .947; area under the curve: 0 μg mL^−1^ mean = 1.533, 20 μg mL^−1^ mean = 1.883, t(57) = 1.244; duration: 0 μg mL^−1^ mean = 26.545 s, 20 μg mL^−1^ mean = 23.994 s, t(57) = −1.051; rise time: 0 μg mL^−1^ mean = 6.860 s, 20 μg mL^−1^ mean = 5.574 s, t(57) = −1.177; decay time: 0 μg mL^−1^ mean = 9.160 s, 20 μg mL^−1^ mean = 9.569 s, t(57) = 0.369; Figure 5f). While some nanomaterials have been shown to alter calcium signaling dynamics in some instances, the exact mechanisms are not fully understood. Graphene oxide (GO) exposure has been shown to change intracellular Ca^2+^ homeostasis and impair spontaneous and evoked signals in primary rat astrocytes.^[49]^ Similarly, exposure of A549 cells to carbon black nanoparticles disrupts calcium homeostasis via ROS generation, suggesting that oxidative stress may contribute to nanomaterial-induced Ca^2+^ signaling dysregulation.^[50]^ Our imaging approach primarily captured somatic Ca^2+^ transients, whereas astrocytic processes display distinct, highly localized calcium dynamics.^[51]^ Investigating these finer-scale dynamics could provide deeper insight into the potential functional effects of Ti_3_C_2_T_x_ exposure. Future studies should also evaluate any dose-dependent and aggregation effects on baseline activity.

## 3. Conclusion

This study provides the first systematic evaluation of Ti_3_C_2_T_x_ MXene–astrocyte interactions, addressing a critical gap in the literature and extending MXene research to glial cells. Astrocytes play an active role in neuronal communication, synapse formation, and injury response, making their interactions with materials like Ti_3_C_2_T_x_ MXene particularly relevant for modulating distinct biological processes. Understanding these interactions is essential for developing neuroelectronic devices that integrate with and modulate the activity of both neuronal and glial cells. Our findings demonstrate astrocyte biocompatibility with Ti_3_C_2_T_x_, laying the groundwork for future glial-targeted bioelectronic interfaces and neuromodulation strategies. Phase contrast and SEM imaging confirmed Ti_3_C_2_T_x_ adhesion to the astrocyte membranes, while viability assays demonstrated robust astrocyte survival across all tested conditions. Morphological analysis showed no significant structural changes. Calcium imaging revealed spontaneous Ca^2+^ activity in both control and Ti_3_C_2_T_x_-treated cultures, with no significant differences in event frequency or kinetics, supporting the functional biocompatibility of Ti_3_C_2_T_x_ flakes under these conditions. Further studies will be needed to explore potential dose-dependent or long-term effects, as well as localized signaling dynamics in astrocytic processes.

Beyond their well-established neuronal applications, MXenes are emerging as versatile materials for a range of biomedical technologies, including biosensors, tissue engineering scaffolds, immunotherapy strategies, and advanced bioelectronic devices.^[23]^ The efficient photothermal properties of Ti_3_C_2_T_x_ have already enabled its use in targeted cancer treatments and remote, non-genetic optical neurostimulation of neurons.^[22,52]^ Given these expanding applications, Ti_3_C_2_T_x_ MXene demonstrates strong potential for integration into cutting-edge bioelectronic interfaces and as a tool for selective modulation of astrocyte activity for therapeutic applications. However, to fully realize the potential of Ti_3_C_2_T_x_ MXene in physiological environments, further studies are necessary to investigate how MXene aggregation influences its stability, dispersibility, and cellular interactions over longer timescales. While this study provides a comprehensive baseline assessment of astrocyte responses to Ti_3_C_2_T_x_ MXene flakes, a more detailed exploration of molecular markers associated with astrocyte function and ion homeostasis may be warranted in future studies. Given the inherent limitations of 2D in vitro models, these follow-up studies may benefit from more physiologically relevant systems, such as 3D cultures or in vivo models, where astrocyte polarization and complex cellular interactions can be more accurately replicated. These could be essential to determine how the spatial and mechanical cues of the brain microenvironment influence cellular responses to Ti_3_C_2_T_x_.

The current study underscores the biocompatibility of Ti_3_C_2_T_x_ flakes with astrocytes while providing a foundation for further studies to assess the potential for MXene-based nanomaterials in neural interfaces. The potential of MXenes to serve as active components in bioelectronic systems extends beyond their neuronal applications, positioning them as promising candidates for glial-targeted therapeutic applications. Future advancements in the field could drive nanomaterial-based innovations in neuroelectronic interfaces for precise neuromodulation and treatment of neurological disorders.

## 4. Experimental Section

### Cell Culture:

Primary cerebral cortical astrocytes were isolated from postnatal day 0 (P0) Sprague–Dawley neonatal rat pups (Charles River Laboratories) using methods adapted from previously reported studies.^[53]^ Both male and female pups were used without sex-based selection. All animal procedures were approved by the Institutional Animal Care and Use Committee (IACUC) of the University of Pennsylvania under protocol number 0 1865 to minimize animal distress. Briefly, cortices were dissected, and the meninges were removed. The tissue was minced and enzymatically dissociated with 5 mL 0.25% trypsin-EDTA per two pup brains for 5–7 min at 37 °C. 400 μL DNase (0.1 mg mL^−1^) was added per two brains, and the cells were triturated with a pipette and centrifuged at 1800 g for 3 min. The cell pellet was then resuspended in 10 mL DMEM/F12 supplemented with 10% fetal bovine serum (FBS) and 1% Penicillin-Streptomycin and incubated in T75 flasks at 37 °C with 5% CO_2_ for expansion. Astrocyte cultures were generated using well-established protocols involving mechanical agitation and selective passaging to enrich for astrocytes.^[53–55]^ Prior to media changes, cultures were mechanically agitated to dislodge and remove less adherent cell types, suchas microglia and oligodendrocyte precursors. Cultures were passaged at least four times upon reaching ≈90% confluency to ensure the maturity of the astrocyte phenotype and yield nearly pure astrocyte populations (*>*95%). Media was changed at 24 h after plating and then every 2–3 days until cells reached ≈90% confluence, at which point they were passaged using 0.25% trypsin-EDTA and incubated for 5–7 min to allow cell detachment. The trypsinization reaction was then quenched by adding serum-containing medium, and cells were centrifuged at 1800 g for 3 min, resuspended in fresh medium, and split in a 1:10 ratio into new T75 flasks for continued expansion. Astrocytes between passages 4–8 were plated into tissue culture plastic well plates or onto sterile glass coverslips coated with 20 μg mL^−1^ poly-L-lysine (for high-magnification imaging) using Neurobasal medium supplemented with 2% B27, 1% G5, 0.25% L-glutamine, and 1% Penicillin-Streptomycin. The chemically defined serum-free formulation was used for experimental consistency and to minimize external influences during the experiments, as described in previous studies.^[55,56]^

### Plating Astrocytes on Glass Coverslips Coated with Poly-L-Lysine (PLL):

Sterile 20 mm round glass coverslips (229 173, CellTreat, MA, USA) were used for plating astrocytes in 12-well plates. Coverslips were placed into each well with sterile forceps and pre-coated with 20 μg mL^−1^ PLL solution, ensuring the coverslips were fully submerged. Following overnight incubation, the PLL solution was aspirated, the coverslips were washed three times with sterile cell culture-grade water, and the coverslips were allowed to dry completely in a laminar flow hood for ≈30 min. Once dry, astrocytes were plated directly onto the coverslips as described in the Cell Culture section. At terminal time points, the cultures were fixed in 4.0% paraformaldehyde in 1X phosphate-buffered saline (PBS) for 35 min at room temperature and processed for immunocytochemistry (ICC) and high-magnification imaging, as described in the following sections. For SEM experiments, 1.5 mm-thick glass coverslips with 12 mm diameter coated with PLL were used (GG-12–15-PLL, Neuvitro Corporation, WA, USA), as described in the SEM section.

### Ti_3_C_2_T_x_ MXene Characterization:

Aqueous dispersions of 20 mg mL^−1^ Ti_3_C_2_T_x_ MXene were produced and provided by MuRata Manufacturing, Co., Ltd. Large Ti_3_C_2_T_x_ flakes (≈1.3 μm lateral size) were synthesized using the minimally intensive layer delamination (MILD) method.^[57]^ Raman spectroscopy was performed using a Horiba Raman and AFM system with a 785 nm excitation laser (100x objective, 25% ND filter). Raman spectra of Ti_3_C_2_T_x_ MXene on a glass slide were acquired for 10 s per scan and averaged across 15 scans. Dynamic Light Scattering (DLS) was used to approximate MXene particle size. Briefly, 10 μg mL^−1^ Ti_3_C_2_T_x_ MXene was aliquoted into a cuvette, and particle size was measured using a Zetasizer Nano ZS equipped with an MP2 autotitrator (Malvern Panalytical).

### Ti_3_C_2_T_x_ MXene Treatment of Astrocytes:

Ti_3_C_2_T_x_ suspensions were sterilized under UV radiation for 1 h, diluted in cell culture-grade water to desired concentrations, and stored at 4 °C until use. Astrocytes were treated with MXene flakes at concentrations of 1, 4, and 20 μg mL^−1^, and treatments lasted for either 1 or 10 days in vitro (DIV).

### Live/Dead Assay:

Cell viability was assessed using a Live/Dead viability kit (L3224, Thermo Fisher Scientific, USA). This assay was selected over metabolic assays such as AlamarBlue due to concerns that Ti_3_C_2_T_x_ MXene flakes may catalyze reduction reactions independent of cell viability, potentially confounding outcomes. Astrocytes were incubated with 2 μm Hoechst 33 342, 2 μm Calcein AM, and 4 μm EthD-1 for 30 min at 37 °C according to the manufacturer protocol. Fluorescent images were acquired, and cell viability (%) was calculated as the percentage of live cells relative to the total cells: *Viability (%)* = *(Live Cells/Total Cells)* × *100*. Total cells were counted based on Hoechst staining, and live cells were calculated as the difference between total and dead cells.

### Immunocytochemistry (ICC):

Astrocytes were fixed with 4% paraformaldehyde in 1X PBS for 35 min and permeabilized with 0.1% Triton X-100 and 4% natural horse serum in PBS for 60 min at room temperature. Cells were blocked with 4% horse serum in PBS and incubated overnight at 4 °C with primary antibodies targeting GFAP (ab4674, Abcam, Cambridge, UK) and plasma membrane (C10045, CellMask Orange Plasma Membrane Marker, Invitrogen, MA, USA). Cells were rinsed five times with PBS, with two quick rinses and the remaining three allowing PBS to sit for 5 min each. Following rinsing, cells were incubated with fluorescent-conjugated secondary antibodies and Hoechst 33 342 for 2 h at room temperature, followed by an additional five rinses with PBS using the same protocol. Upon immunofluorescent staining, samples on glass coverslips were transferred to glass slides with Fluoromount-G Mounting Medium (50–187-88, Invitrogen, MA, USA).

### Phase Contrast and Fluorescence Microscopy:

Phase contrast images were acquired using a Nikon Inverted Eclipse Ti-S microscope with a QImaging QIClick Camera, paired with NIS Elements BR software 6.02.03. Fluorescent images were acquired using a Nikon A1Rsi Laser Scanning Confocal microscope paired with NIS Elements AR 4.50.00. Samples were imaged with a 10x, 20x, or 100x objective (CFI Plan Apo Lambda × 10, n.a. 0.45; × 20, n.a. 0.75; × 100 Oil, n.a. 1.45).

### Scanning Electron Microscopy (SEM):

Astrocyte cultures on glass coverslips with PLL coating (GG-12–15-PLL, Neuvitro Corporation, WA, USA) were treated with 0 and 20 μg mL^−1^ Ti_3_C_2_T_x_ for 10 days in vitro and prepared for scanning electron microscopy (SEM) as follows. Samples were fixed overnight with 2% glutaraldehyde in 50 mm Na-cacodylate buffer (pH 7.3) and washed three times with 50 mm Na-cacodylate buffer. Fixed samples were dehydrated in a graded ethanol series over 2.5 h, with three changes of 100% ethanol, followed by 20 min of incubation in 50% hexamethyldisilazane (HMDS) in ethanol, three changes of 100% HMDS (Sigma-Aldrich), and overnight air-drying as previously described.^[58]^ Dried samples were mounted on stubs and sputter-coated with gold/palladium (Au/Pd). SEM experiments were performed at the CDB Microscopy Core (Perelman School of Medicine, University of Pennsylvania) using a Quanta 250 FEG scanning electron microscope (FEI, Hillsboro, OR, USA) at 10 kV accelerating voltage.

### Cell Morphology Quantification:

To analyze astrocyte morphology, a custom image analysis pipeline was implemented for 3D reconstruction and segmentation of cell bodies and nuclei. The analysis involved four primary steps: image processing, cell reconstruction, cell segmentation, and morphology analysis (Figure 4a). Astrocytes were cultured, fixed, immunostained, and imaged as described in previous sections. Fluorescence confocal microscopy images of CellMask-stained astrocytes were preprocessed using ImageJ and Python scripts to adjust brightness and contrast, apply Gaussian blurring, and threshold images for cell and nucleus detection. Down-sampling and 3D rendering were applied to generate volumetric reconstructions of astrocytes. Density-based clustering (DBSCAN) and volumetric filtering were then used to segment individual cells and nuclei. Cells with volumes below 50 units (≈12 μm^3^) and nuclei below 5 units (≈1 μm^3^) were considered non-cell fragments and excluded from analysis. The final segmentation matched nuclei to corresponding cell bodies for subsequent morphometric analysis. The morphological parameters quantified included aspect ratio, circularity, centroid distance, solidity, and ramification. Aspect ratio was defined as the ratio of the maximum Y range to the maximum X range of the cell. Circularity was calculated as C = 4*π*A/P^2^, where A is the projected area and P is the perimeter of the cell body.^[59]^ To provide a consistent basis for comparison, an ellipse was fitted to the projected cell area, with the ellipse dimensions derived from the cell’s maximum X- and Y-ranges. The area and perimeter of the fitted ellipse were calculated as A_ellipse_ =*π*·X·Y and P_ellipse_ = 2*π*[(X^2^+Y^2^)/2]^1/2^. Centroid distance was defined as the Euclidean distance between the centroids of the cell body and nucleus. Cell solidity (or density) was calculated as the ratio of the convex hull volume to the cell body volume.^[40]^ The convex hull refers to the smallest convex polygon that completely encloses all points on the boundary of a cell. Ramification was calculated as *R* = (perimeter/area)/[2(*π*/area)^1/2^], which normalizes the ratio of the cell’s perimeter to its area against the same ratio for a perfect circle of the same area.^[41]^ A higher ramification index indicates increased contour complexity or irregularity compared to a smooth circular shape. The ramification for each cell was determined from the 2D projection with the maximum area, and the perimeter was derived from the convex hull of this projection. To ensure data accuracy and reliability, cells were excluded based on predefined quality control criteria to ensure data accuracy and reliability. Cells with abnormal boundaries, extreme or negative dimensions, or inconsistent 3D reconstructions were manually inspected and removed by an assistant blinded to the experimental condition. Cells lacking a corresponding nucleus or with centroid distances exceeding 100 units (≈62 μm) were excluded, as well as significant outliers using Grubbs’ test.

### Calcium Imaging and Analysis:

Astrocytic calcium dynamics were assessed using Calbryte 520 AM (AAT Bioquest), a cell-permeable calcium-sensitive dye. A 2 mm stock was prepared by dissolving 100 μg in 45.8 μL anhydrous DMSO, aliquoted, and stored at −20 °C until use. For live-cell imaging, astrocytes were incubated with 5 μm Calbryte 520 AM in prewarmed media for 45 min, then washed with fresh media. Imaging was performed on a Nikon Eclipse Ti confocal microscope with a pco.edge 4.2 LT sCMOS camera and Nikon Elements AR 6.10.01 software (Nikon Instruments), capturing 10 frames per second over a 5-min recording period (2048 × 2044 pixels, 1331.2 × 1328.6 μm field of view) in a temperature and CO_2_-controlled stage-top incubator. Each condition (0 v 20 μg mL^−1^ Ti_3_C_2_T_x_ MXene) was assessed across *n* = 3 independent experiments, each conducted on a different day using astrocytes from separate neonatal isolations to ensure biological variability. 42 unique wells per condition were imaged (one video per well), for a total of 84 videos across both conditions. Time-lapse data were acquired in.nd2 format, converted to.tif, and downsampled for computational efficiency. Astrocyte segmentation was performed by generating binary masks using maximum intensity projections and Otsu’s thresholding to differentiate signal from background. Morphological operations (binary dilation, hole-filling) refined the mask, and small debris were removed using connected component analysis. Masks were validated against raw images. Fluorescence intensity was extracted from a circular ROI with a 5-pixel radius around manually annotated cell coordinates, with background fluorescence estimated from non-cellular regions (i.e., pixels outside the mask). Fluorescence traces (ΔF/F_0_) were calculated as (F_t_−F_0_)/ F_0_, where F_0_ was the mean background fluorescence at each time point. Traces were filtered with a 6^th^-order Butterworth low-pass filter (1 Hz cutoff) to reduce noise and a 2^nd^-order Butterworth high-pass filter (0.01 Hz cutoff) to remove global low-frequency trends. Calcium transients were detected using zero-crossing identification and a fixed threshold of 0.1. Local minima refined event boundaries, and events were aggregated per region of interest (ROI). All image processing was performed in Python 3.10.13.

### Statistical Analysis:

All data were analyzed using Prism 10 (GraphPad Software, USA) or custom Python 3.10.13 scripts. Data are presented as mean ± standard error of the mean (SEM), unless otherwise specified. Statistical significance (*p* < 0.05) is denoted with (*). For viability assays, statistical comparisons across treatment groups were performed using one-way analysis of variance (ANOVA) with Tukey’s post hoc test to correct for multiple comparisons (*α* = 0.05). Each condition included *n* = 3 biological replicates, with 3 technical replicates per condition and ≥4 randomly selected imaging fields per replicate. For morphology analyses, comparisons between control and treated groups were made using two-tailed unpaired t-tests (*α* = 0.05) for each parameter (aspect ratio, circularity, centroid distance, solidity, ramification). Outlier exclusion was performed using Grubbs’ test (*α* < 0.05) prior to analysis. Morphological data were pooled from n = 45 confocal images per condition, with an average of 91 segmented cells per condition. For calcium imaging, the proportion of cells that were active, the event rate distribution across active cells, and event features including the amplitude, area under the curve, duration, rise time, and decay time were examined. Proportion of active cells was computed for each well, and differences across conditions were tested using Student’s two-sample t-test (scipy.stats.ttest_ind; equal_var set to “True”). Event rate distribution was computed by condition and analyzed with a Kolmogorov-Smirnov test (scipy.stast.ks_2samp). Event features were tested using Student’s two-sample t-test (n = 154 events for 0 μg mL^−1^; n = 141 events for 20 μg mL^−1^ condition). Reported t-values and degrees of freedom were not adjusted for multiple comparisons.

## Supplementary Material

videoS1

SuppMat

Supporting Information

Supporting Information is available from the Wiley Online Library or from the author.

## Data Availability

The data that support the findings of this study are available from the corresponding author upon reasonable request.
