# Supplementary material for: Astrocyte Interactions With Ti3C2Tx MXene Flakes: Insights Into Viability, Morphology, and Functionality
Source: Adv Mater Interfaces. Author manuscript; Available in PMC 2025 Dec 30. (PMC12747572; doi:10.1002/admi.202500261)
Supplement: SuppMat [file NIHMS2126595-supplement-SuppMat.pdf]

ADVANCED MATERIALS INTERFACES
Open Access

Supporting Information

for *Adv. Mater. Interfaces*, DOI 10.1002/admi.202500261

Astrocyte Interactions With $Ti_3C_2T_x$ MXene Flakes: Insights Into Viability, Morphology, and Functionality

Dimitris Boufidis, Elizabeth N. Krizman, Cybelle M. Smith, Yihan Xie, Raghav Garg, John C. O'Donnell, Flavia Vitale and D. Kacy Cullen**

Supporting Information

Astrocyte Interactions with $Ti_3C_2T_x$ MXene Flakes: Insights into Viability, Morphology, and Functionality

Dimitris Boufidis, Elizabeth N. Krizman, Cybelle M. Smith, Yihan Xie, Raghav Garg, John C. O'Donnell, Flavia Vitale, D. Kacy Cullen**

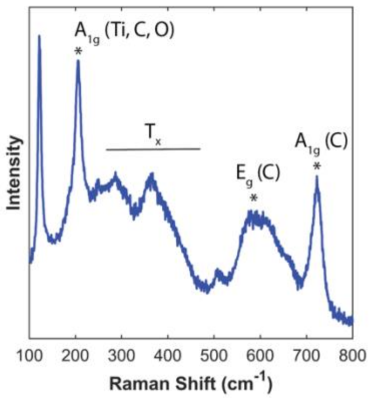

Figure S1. Raman spectrum of 2D $Ti_3C_2T_x$ MXene flakes confirming their structural and chemical composition. The peak at ~200 cm^{-1} corresponds to out-of-plane vibrations involving titanium, carbon, and oxygen atoms. The peaks between 230 and 480 cm^{-1} reflect in-plane vibrations primarily associated with oxygen-containing functional groups. Higher frequency peaks at ~600 and ~730 cm^{-1} are linked to vibrations of carbon atoms within the $Ti_3C_2T_x$ structure[32, 33].

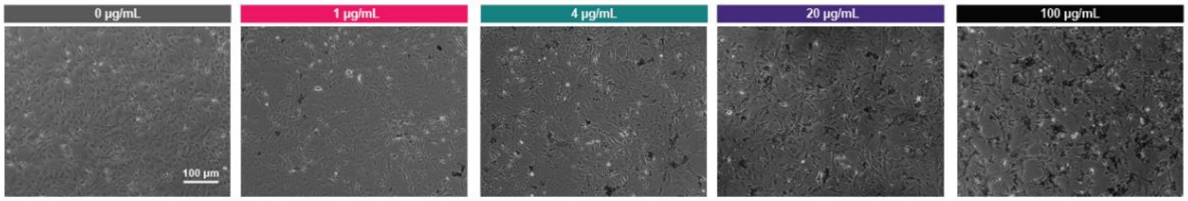

Figure S2. Phase Contrast images of rat astrocyte cultures treated with 0, 1, 4, 20, and 100 µg/mL $Ti_3C_2T_x$ for 10 days *in vitro*.

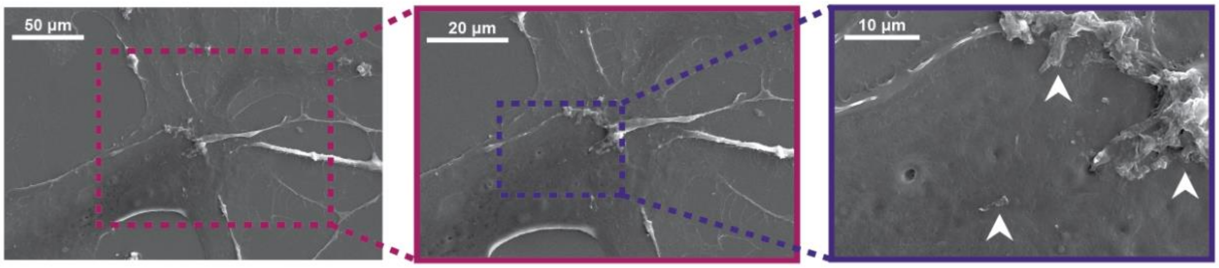

Figure S3. SEM images of 1,000× (left) and 5,000× (right) magnification confirm that the $Ti_3C_2T_x$ flakes of **Error! Reference source not found.**. Scanning Electron Microscopy (SEM) of astrocytes cultured with $Ti_3C_2T_x$ for 10 days in vitro. **(a)** Representative 200× SEM images showing untreated (0 µg/mL, left) and $Ti_3C_2T_x$-exposed (20 µg/mL, right) astrocytes. White circles highlight $Ti_3C_2T_x$ flakes and aggregates. **(b)** SEM at 2,500x magnification showing $Ti_3C_2T_x$ flakes firmly adhering to the astrocyte membranes. **(c)** Zoomed-in SEM at 5,000× of the highlighted region in **(b)**. SEM micrograph of **(d)** 5,000× and **(e)** 20,000× magnification of $Ti_3C_2T_x$ on the astrocyte membrane. are on the astrocyte membrane and not on the SEM sample substrate.

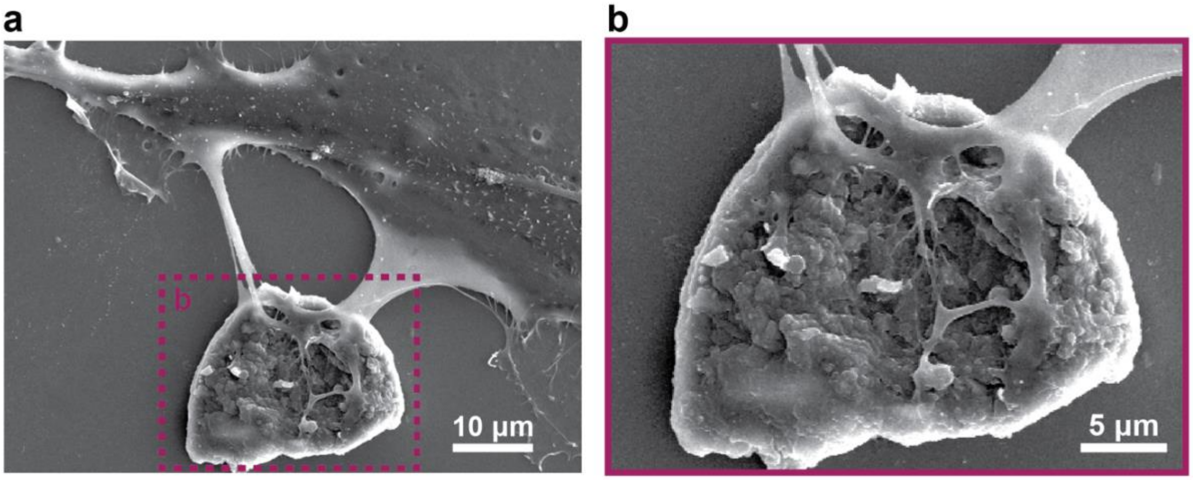

Figure S4. (a-b) SEM micrographs of astrocytes interacting with a large $Ti_3C_2T_x$ MXene aggregate in the vicinity of a cell. In **(b)**, fine astrocytic processes are observed extending toward and adhering to the aggregate's surface.

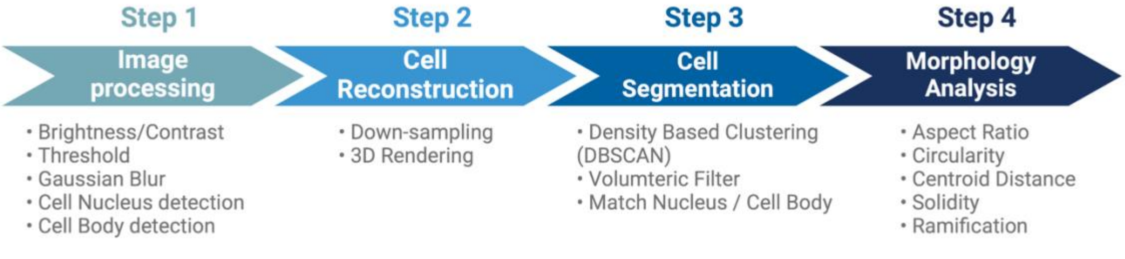

Figure S5. Overview of the morphology analysis pipeline.

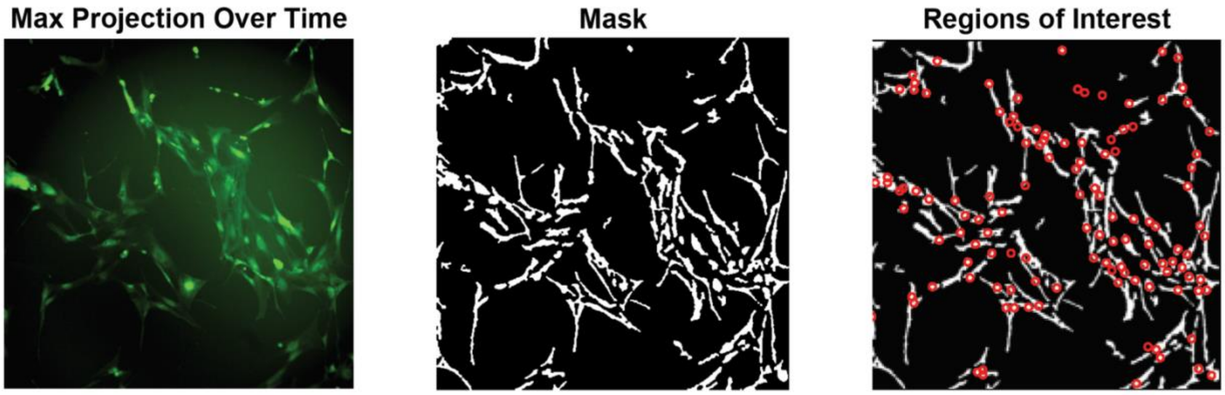

Figure S6. Calcium imaging of astrocytes exposed to $Ti_3C_2T_x$ MXene flakes. Representative calcium signaling data showing (left) the maximum projection of fluorescence over time, (center) a segmentation mask for detected astrocytes, and (right) identified regions of interest (ROIs) used for calcium event analysis.
